# μ-Opioid Receptor-Mediated AT1R–TLR4 Crosstalk Promotes Microglial Activation to Modulate Blood Pressure Control in the Central Nervous System

**DOI:** 10.3390/antiox10111784

**Published:** 2021-11-08

**Authors:** Gwo-Ching Sun, Jockey Tse, Yung-Ho Hsu, Chiu-Yi Ho, Ching-Jiunn Tseng, Pei-Wen Cheng

**Affiliations:** 1 Department of Anesthesiology, Kaohsiung Veterans General Hospital, Kaohsiung 813414, Taiwan; gcsun39@kmu.edu.tw; 2 Department of Anesthesiology, Kaohsiung Medical University Hospital, Kaohsiung 80708, Taiwan; 1030402@kmuh.org.tw (J.T.); 1020366@ms.kmuh.org.tw (Y.-H.H.); 3 Department of Anesthesiology, School of Medicine, College of Medicine, Kaohsiung Medical University, Kaohsiung 80708, Taiwan; 4 Department of Medical Education and Research, Kaohsiung Veterans General Hospital, Kaohsiung 813414, Taiwan; cyho@vghks.gov.tw (C.-Y.H.); cjtseng@vghks.gov.tw (C.-J.T.); 5 Department of Biomedical Science, National Sun Yat-Sen University, Kaohsiung 80424, Taiwan

**Keywords:** angiotensin II type 1 receptor (AT1R), hypertension, heterodimer, toll like receptor 4, nucleus tractus solitarii, opioids

## Abstract

Opioids, a kind of peptide hormone involved in the development of hypertension, cause systemic and cerebral inflammation, and affects regions of the brain that are important for blood pressure (BP) control. A cause-and-effect relationship exists between hypertension and inflammation; however, the role of blood pressure in cerebral inflammation is not clear. Evidence showed that AT1R and μOR heterodimers’ formation in the NTS might lead to the progression of hypertension. In this study, we investigated the formation of the μOR/AT1R heterodimer, determined its correlation with μORs level in the NTS, and explored the role of TLR4-dependent inflammation in the development of hypertension. Results showed that Ang II increased superoxide and Iba-1 (microgliosis marker: ionized calcium-binding adaptor molecule (1) levels in the NTS of spontaneously hypertensive rats (SHRs). The AT1R II inhibitor, losartan, significantly decreased BP and abolished superoxide, Iba-1, TLR4 expression induced by Ang II. Furthermore, losartan significantly increased nNsOS^S1416^ phosphorylation. Administration of a μOR agonist or antagonist in the NTS of WKY and SHRs increased endogenous μ-opioids, triggered the formation of μOR/AT1R heterodimers and the TLR4-dependent inflammatory pathway, and attenuated the effect of depressor nitric oxide (NO). These results imply an important link between neurotoxicity and superoxides wherein abnormal increases in NTS endogenous μ-opioids promote the interaction between Ang II and μOR, the binding of Ang II to AT1R, and the activation of microglia. In addition, the interaction between Ang II and μOR enhanced the formation of the AT1R and μOR heterodimers, and inactivated nNOS-derived NO, leading to the development of progressive hypertension.

## 1. Introduction

Hypertension poses a significant health problem and intensive efforts have been made to elucidate its underlying mechanisms [1]. Approximately one-third of adults have hypertension in the United States and 50% of hypertensive patients show satisfactory results after treatment [2]. The major reason is that most of the existing anti-hypertensive therapies target peripheral mechanisms and are not effective for treating hypertension driven in the central nervous system (CNS) [2]. A better understanding of the CNS mechanisms underpinning the pathogenesis of hypertension can lead to the discovery of novel strategies for the prevention and treatment of hypertension.

The nucleus tractus solitarius (NTS), located in the dorsal medulla of the brainstem, is primarily responsible for integration of cardiovascular (CV) regulation and other autonomic functions of the central nervous system (CNS). In the presence of noxious stimuli, a phenomenon known as “hypertensive hypoalgesia” [3], which is related to decreased pain sensitivity [4], occurs as a result of a homeostatic feedback loop caused by BP stabilization. Numerous neuropeptides such as opioids and angiotensin are implicated in the CV system [5,6]. The renin–angiotensin system (RAS) is an enzyme neuropeptide system in the brain and periphery that has been well-studied, and has served as a neuronal model for peptide regulation. Angiotensin II (Ang II) is a primary effector peptide of the renin–angiotensin–aldosterone system (RAAS) which binds to the G-protein-coupled receptor subtypes AT1R and AT2R with similar affinity in multiple cell and tissue types [7]. Elevated circulating Ang II in the brain was reported to be associated with the genesis of arterial hypertension [8], whereas the overactivation of RAAS is crucially involved in the pathogenesis of hypertension and hypertension-related cardiovascular disorders [9]. Moreover, the beneficial effects of previous studies have shown that RAS blockers can reduce the development of hypertension and its associated neuropathic pain, cognitive impairment and cerebral injury [6,10,11]. By understanding role of the neuropeptides, opioids and angiotensin in CV function, we hope to trace the molecular origin of heart failure during the development of hypertension.

Other experimental evidence has demonstrated significant functional overlapping of RAS components and endogenous opioids (alongside their receptors) in the brain and periphery regions, showing synergistic interaction between angiotensin and opioids [12]. Angiotensin increases opioid levels to induce polydipsia, analgesia, LH secretion and hypertension, which are abolished in the presence of an opioid antagonist, namely naloxone. On the other hand, opioids increase angiotensin II levels by activating renin and angiotensin-converting enzyme (ACE) either directly or indirectly [12]. Furthermore, opioid-induced increases in ACE activity may trigger a negative feedback mechanism that affects the influence of opioids, thereby enhancing the metabolism of endogenous opioids through neutral endopeptidases and dipeptidylcarboxypeptidase [13].

Previous experiments suggest that hypertension is characterized by pro- and antioxidant mechanisms [14], inflammatory disorders [15], GPCR heterodimers [16], and sympathetic/parasympathetic tone imbalances [17]. Accumulating evidence suggests that the Ang II-AT1R axis stimulates innate and adaptive immune systems [18,19,20]. The blockade or knockdown of toll-like 4 receptor (TLR4), which is required for integral sensing and signaling of the innate system, attenuates Ang II-dependent hypertension, as well as renal and cardiac injury [19]. Nair et al., proposed that Ang II stimulates the AT1R to release high-mobility group protein 1 (HMBG1), a ligand required for TLR4 to evoke inflammation [21]. Direct stimulation of the MD2-TLR4 complex by Ang II is clinically important as Ang II receptor blockers (ARBs) are capable of increasing Ang II through the inhibition of renin release [22]. Thus, the concern is that ARB treatment may cause the unintended consequence of stimulating TLR4-dependent inflammation. This mechanism may potentially diminish the optimal effects of ARBs in the treatment of cardiovascular disease (CVD) [23]. Horvath et al. previously reported that morphine administration results in changes in the microglia and astrocytes, as well as increased cellular hypertrophy, microglial CD11b, Iba1 expression and astrocytic GFAP expression in vitro [24] and in vivo [25]. On the contrary, the inhibition of microglial P2X4 receptors attenuates morphine tolerance, and Iba1, GFAP and μ opioid receptor protein expression [26].

SHRs were used because they show high Ang II and AT1R levels compared tok WKY [27]. During Ang II-induced hypertension, peripheral Ang II infusion increased ROS production and brain inflammation [28]. Evidence indicates that Ang II stimulates the innate system by direct activation of TLR4 through an AT1R-independent mechanism [29]. Opioids increase angiotensin II levels, either directly or indirectly, by activating renin and ACE [12], and opioids are known to interact with RAS components. A previous report showed that WKY infused with AngII showed increases in BP, and increased AT1R and μOR heterodimers, in the NTS [6], prompting the need to understand the role of μOR in the formation of AT1R-μOR heterodimers and the mechanism of microglial and TLR4 activation. However, the molecular mechanism of this process remains unclear. This study aimed to determine: (1) the interplay between μORs and AT1R in the formation of heterodimers in the NTS when endogeneous opioids are present; (2) the function of Ang II in TLR4-dependent inflammation during high BP in the NTS; and (3) the major factor that leads to the formation of μOR/AT1R heterodimers in the NTS. Our results demonstrated that an increase in endogenous μ-opioids in the NTS induced the formation of μOR/AT1R heterodimers and the TLR4-dependent inflammatory pathway, which attenuated the NO-dependent depressor effect. In summary, endogenous increases in μ-opioid are most likely factor to contribute to the pathogenesis of hypertension through the AT1R–TLR4 axis.

## 2. Materials and Methods

### 2.1. Animals

Animal studies were conducted in compliance with the Animal Research: Reporting of In Vivo Experiments (ARRIVE) guidelines as described previously [30,31]. All protocols were approved by Animal Research Committee and the institutional review board at VGHKS (VGHKS-2021-2023-A009; VGHKS-2020-2022-A046) and an affidavit of approval was obtained in with the animal care protocol of Kaohsiung Medical University (109087). Wistar-Kyoto rats (WKY) and SHRs were obtained from the National Science Council Animal Facility (NSCAF; Taipei, Taiwan), and housed in an animal facility at Kaohsiung Veterans General Hospital (VGHKS; Kaohsiung, Taiwan). NSCAF and VGHKS were approved by the Association for Assessment and Accreditation of Laboratory Animal Care (AAALAC). WKY were caged under specific pathogen-free (SPF) conditions at VGHKS, and were free of infectious and pathogenic organisms capable of interfering with research subjects. The WKY were caged in individual cages, provided 12-h light-and-dark cycle, and kept at a temperature between 23 and 24 °C. Animals were provided with normal rat chow (Purina, St. Louis, MO, USA) and tap water ad libitum. The animals were settled in the housing environment for one week for adjustment before being habituated to the indirect blood pressure measurement for another week. The highest and lowest datum were excluded, the remaining six data were averaged for each group (*n* = 6). The SBP of the rats was measured before the start of the WKY, SHR, and losartan treatments (week 0) using a tail-cuff monitor (Noninvasive Blood Pressure System, SINGA, Taipei, Taiwan). The rats were placed in the fixer for 30 min at a constant temperature of 37 °C. During measurement, six individual readings were obtained. The highest and lowest readings were discarded, and the averages of the remaining eight readings were obtained. The SBP was measured at the same time on a daily basis. The rats were randomly assigned to five groups, with six in each group—(1) WKY: 6 wo WKY; (2) SHR: 6 wo SHR; (3) WKY: 20 wo WKY; (4) SHR: 20 wo SHR (saline); (5) SHR + losartan: 20 wo SHR + losartan (week-old abbreviated as wo). Losartan (30 mg/kg per day) was administered by gavage to 20 wo SHRs for 2 weeks. Animals were euthanized using 100% CO_2_, a procedure that was in accordance with the 2013 American Veterinary Medical Association (AVMA) guidelines.

### 2.2. Intra-NTS Microinjection

Twenty-week-old WKY were anesthetized using urethane (1.0 g kg^−1^ intraperitoneally (i.p.), supplemented with 300 mg kg^−1^ intravenously (i.v.) when required). Blood pressure and heart rate were measured via femoral-artery cannula using a pressure transducer and polygraph (Gould, Cleveland, OH, USA), and a tachograph preamplifier (Gould, Cleveland, OH, USA), respectively. Tracheostomy was performed to maintain airway patency. For brainstem nuclei microinjection, animals were placed in a stereotaxic instrument (Kopf, Tujunga, CA, USA), with the head positioned 45°downward to expose dorsal surface of the medulla with limited craniotomy, followed by a 1h resting period. Single-barrel glass catheters (0.031-inch outer diameter (OD), 0.006-inch internal diameter (ID); Richland Glass Co, Vineland, NJ, USA) with external tips of 40 μm in diameter were used. L-glutamate (0.154 nmol 60 nL^−1^) was microinjected to induce the characteristic decrease in BP (BP ≥ −35 mmHg), in order to verify that needle tip was located in the medial site, in one-third of the NTS. The precise coordinates were as follows: anteroposterior, 0.0 mm; mediolateral, 0.5 mm; and vertical, 0.4 mm (with the obex as a reference) [32]. For microinjections, 0.3 nmol of the DAMGO μ opioid agonist (Sigma, St. Louis, MO, USA), or 0.3 nmol of the guanfacine α2A agonist (Sigma, St. Louis, MO, USA), were prepared in 0.9% saline for use.

### 2.3. In Situ Detection of Superoxide in the NTS

Endogenous in vivo superoxide production in the NTS was determined via dihydroethidium staining (DHE; Invitrogen, Carlsbad, CA, USA). The NTS was dissected, quickly frozen, embedded in OCT, and immersed in liquid nitrogen. Cryostat slices (30 μm) were stained with 1 μM DHE in the dark for 30 min at 37 °C. The samples were analyzed using a confocal microscope (Carl Zeiss LSM 5 PASCAL, Göttingen, Germany).

### 2.4. Immunofluorescence Staining Analysis

The rats were perfused using 0.9% saline and 4% formaldehyde, followed by 30% sucrose solution. Brainstems were cut into 20 μm-thick sections, incubated in anti-endomorphin-2, anti-IBA1, anti-AT1R (ab124505) and anti-nNOS^S1416^ primary antibodies at a dilution ratio of 1:100. After washing with PBS, sections were incubated in Alexa Fluor 488 or 588-conjugated donkey anti-rabbit IgG (1:200; Invitrogen, Carlsbad, CA, USA) at 25 °C for 2 h, and analyzed using a fluorescence microscope and Zeiss LSM Image software (Carl Zeiss MicroImaging).

### 2.5. Proximity Ligation Assay (PLA)

The Duolink in situ proximity ligation assay (PLA; OLINK Bioscience, Uppsala, Sweden) was utilized to detect the formation of AT1R/μOR heterodimers. The NTSs of SHRs and WKY were examined to detect the formation of AT1R (sc-515884) and μOR (bs-3623R) heterodimers in situ. The rats were first perfused in saline, then 4% formaldehyde, and finally 30% sucrose solution. Brainstem’s microsections of 5 μm thickness were obtained. Primary antibody diluent (OLINK Bioscience), containing two primary antibodies (1:100 for goat anti-μ receptor antibodies and 1:100 rabbit anti-AT1R antibodies), was added to the sections, and incubated overnight at 4 °C. Next, PLA secondary antibody with specific oligonucleotides, anti-rabbit plus and anti-goat minus (OLINK Bioscience), were applied to the sections and incubated for 2 h at 37 °C. Samples underwent ligation to allow nearby oligonucleotide probe pairs to form closed circles, and signals were amplified in the amplification solution. Images were acquired using a confocal laser scanning microscope (Carl Zeiss LSM 5 PASCAL), and were further processed by LSM 5 PASCAL software (Version 3.5, Carl Zeiss), which automatically counted the number of spots per unit of surface area.

### 2.6. Measurement of NO in the NTS

The NTS (10 mg) was deproteinized using Microcon YM-30 centrifugal filter units (Millipore, Bedford, MA, USA). The total content of NO in the samples was determined through a procedure that is based on the purge system, using the Sievers Nitric Oxide Analyzer (NOA 280i) (Sievers Instruments, Boulder, CO, USA) to assess chemiluminescence. The samples (10 μL) were injected into a reflux column containing 0.1 mol/L VCl_3_ in 1 mol/L HCl at 90 °C to reduce any existing nitrate and nitrite (NOx) to NO. NO reacted with the O_3_ produced by the analyzer to form NO_2_. The resulting emission from the excited NO_2_ was detected by a photomultiplier tube and recorded digitally (mV). The standard curve was determined for the NaNO_3_ concentrations, and NO levels were corrected for the rats’ NTS.

### 2.7. Immunoblotting Analysis

Proteins (20 μg per sample) were quantified using a BCA protein assay (Pierce Chemical Co., Rockford, IL, USA), resolved in 6% polyacrylamide gel, and transferred to the PVDF membrane (GE Healthcare, Buckinghamshire, UK). The membranes were incubated at 4 °C overnight using the following primary antibodies: mouse anti-P-eNOS^S1177^, mouse anti-DDAH1, and mouse anti-eNOS (BD Biosciences, San Jose, CA, USA); mouse anti-actin and mouse anti-nNOS (Millipore); and mouse anti-P-nNOS (Abcam, Cambridge, UK) (dilution at 1:1000).

### 2.8. Statistical Analysis

All measurements were repeated at least three times under independent conditions. The results shown are the mean ± the standard error of the mean (SEM). Statistics were analyzed using the Mann–Whitney U-test. One-way analysis of variance (ANOVA) with Scheffé post-hoc comparison was used to compare differences between groups. SPSS version 20.0 (SPSS Inc, Chicago, IL, USA) was applied for analyzing the raw data. * *p* < 0.05 and ** *p* <0.01 indicate significance.

## 3. Results

### 3.1. Ang II Elevates the ROS-Microglial Activation and Reduces the Systemic Vasodepressor Effect of NO by Impairing the nNOS Pathway in the NTS of Spontaneously Hypertensive Rats

**Figure 1:**
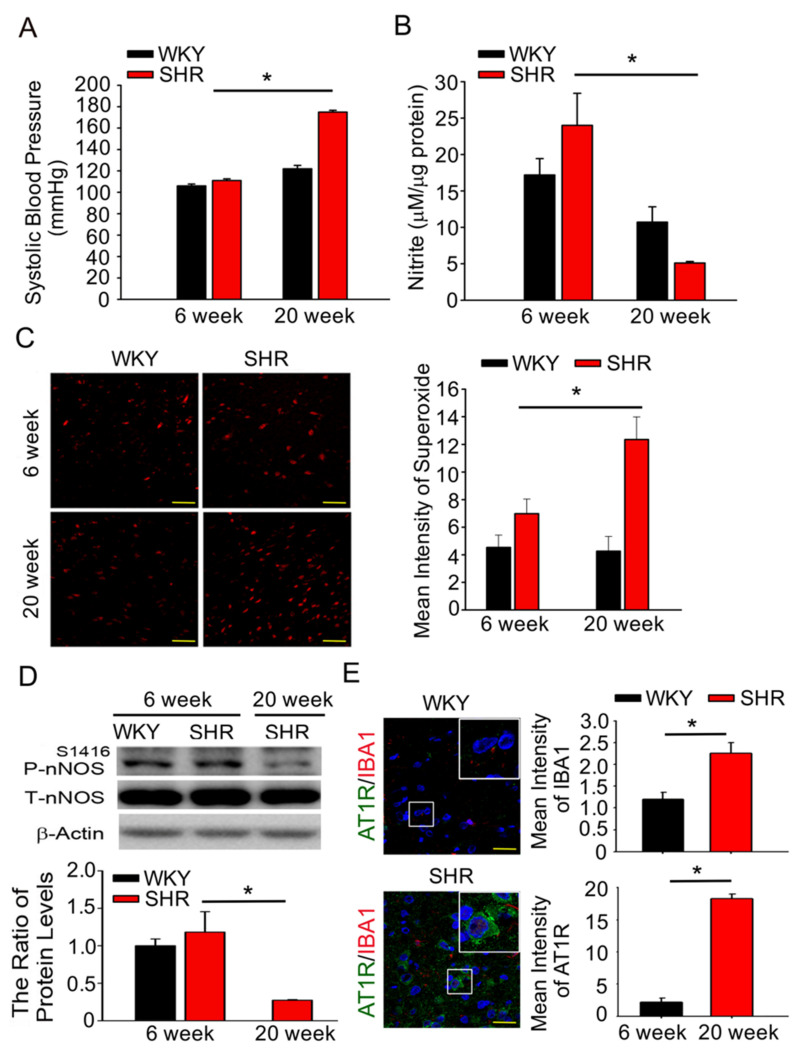
Ang II supports superoxide generation, increases the activation of microglia and restores the nNOS pathway in the NTS of spontaneously hypertensive rats. (**A**,**B**) Graph showing systemic blood pressure (SBP) and nitric oxide (NO) concentrations in the nucleus tractus solitarius (NTS) of normotensive Wistar Kyoto rats (WKY; 6-, 20-week-old) and spontaneously hypertensive rats (SHRs; 6-, 20-week-old). The bar graph shows the NO concentration as μM nitrate per μg of total protein. (**C**) Representative images of DHE-treated brain sections, images photographed at ×280 magnification. ROS index in the NTS of the SHRs groups (20 weeks old) was compared to SHRs (6 weeks old). The ROS index is the relative mean fluorescence intensity for dihydroethidium. Sections including the NTS of SHRs rats displayed significant increase in DHE fluorescence compared with the SHRs (6 weeks old) group sections. (**D**) Quantitative immunoblotting analysis of nNOS^S1416^ phosphorylation in the NTS of 6-week-old WKY, SHRs and 20-week-old SHRs. One-way analysis of variance (ANOVA) with Scheffé post-hoc was performed for statistical analysis in Figure 1A–D. (**E**) Representative images for immunofluorescence staining of AT1R-positive cells (green) and microglial marker IBA-1 (red) in NTS sections of WKY and SHRs, counterstained with 4′,6-diamidino-2-phenylindole (DAPI for blue color). The images were photographed at ×400 and 1000 magnification. The Mann–Whitney U-test was performed for statistical analysis in Figure 1E. The values are presented as mean ± SEM. * *p* < 0.05 indicates significant difference from 6-week-old SHRs (*n* = 6~8 per group).

To determine the effect of ROS-dependent NO release on systemic BP (SBP) in the NTS, we examined the SBP, nitrate, IBA1 level, and ROS production in the NTS of WKY controls, prehypertensive 6 wo SHRs, and hypertensive 20 wo SHRs. A significant age-dependent increase in BP occurred in SHRs between 6 and 20 wo, and NTS NO levels were significantly decreased (*p* < 0.05, *n* = 6; Figure 1A,B). Superoxide levels in the NTS were significantly high in the SHRs between 6 and 20 wo (*p* < 0.05, *n* = 6; Figure 1C). Immunoblotting analyses demonstrated that the phosphorylation of nNOS^S1416^ was significantly decreased in 20 wo SHRs (*p* < 0.05, *n* = 6; Figure 1D). The α2A-AR and μOR heterodimers were determined using PLA. At 6 wo, SHRs and WKY exhibited normal systolic BP, and no differences in the levels of the α2A-AR and μOR heterodimers were observed. Interestingly, the levels of NTS AT1R and IBA1 were significantly elevated in adult SHRs compared to WKY (Figure 1E), indicating that Ang II enhanced ROS production and microglial activation, which impaired the nNOS pathway in the NTS of spontaneously hypertensive rats.

### 3.2. AT1R Inhibitors Decrease BP via Inhibition of AT1R-Induced Superoxide to Enhance Microglia Activity in the NTS

**Figure 2:**
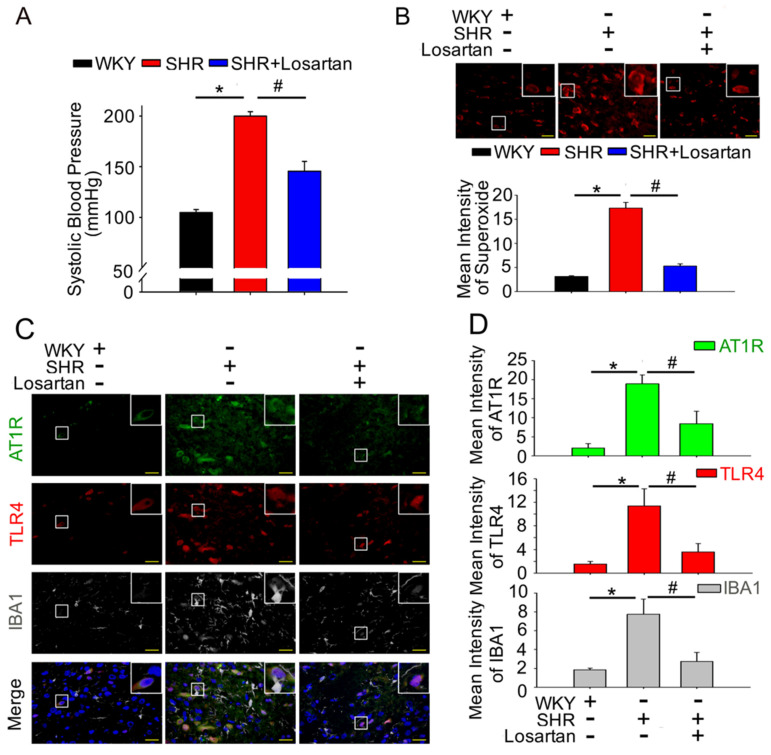
The downregulation of AT1R-induced superoxide generation is associated with the activation of microglia and the expression of TLR4 in the NTS of spontaneously hypertensive rats. (**A**) SBP after losartan administration for 2 weeks. (**B**) Representative images of DHE-treated brain sections, photographed at ×280 magnification. Bar graph representation of ROS index in the NTS of WKY, SHRs, and SHRs treated with losartan. (**C**,**D**) Representative fluorescence images for AT1R (green), TLR4 (red) and microglial marker IBA-1 (white)-positive cells in the NTS sections of WKY, SHRs, and SHRs treated with losartan. The images were photographed at ×400 and 1000 magnification. The Mann–Whitney U-test was performed for statistical analysis in Figure 1E. One-way analysis of variance (ANOVA) with Scheffé post-hoc was performed for statistical analysis. The values are presented as mean ± SEM. * *p* < 0.05 indicates significant difference from 20-week-old WKY. # *p* < 0.05 versus SHR. All data are presented as means ± SEM (*n* = 6 per group).

In this section, we show that the activation of AT1R increased microglia and ROS production in the NTS, which further reduced nNOS^S1416^ phosphorylation during the development of ANG II-induced hypertension. Therefore, we investigated the progression of hypertension after the activation of the microglia and TLR4 resulted from the increase in AT1R, further studied the effects of the AT1R inhibitor losartan on SBP, superoxide production, and activity of the microglia. SBP was found to be significantly lower, and superoxide levels in the NTS were significantly lower in the losartan-treated SHRs as compared to the untreated SHRs (Figure 2A and Figure 4B, lane 2 and lane 3, respectively; ^#^ *p* < 0.05, *n* = 6). Immunofluorescence staining demonstrated that losartan-treated SHRs showed significantly reduced AT1R and TLR4 activation of microglial cells in the NTS (Figure 2C,D, lane 2 and lane 3, respectively; ^#^*p* < 0.05, *n* = 6). These results indicated that AT1R-induced superoxide generation led to an increase in the activation of microglia and microglial TLR4, thereby inducing progressive hypertension.

### 3.3. The Potential Role of μOR in AT1R-Induced Microglia Activation and TLR4 Expression in the NTS Explains Its Function in Cardiovascular Regulation

**Figure 3:**
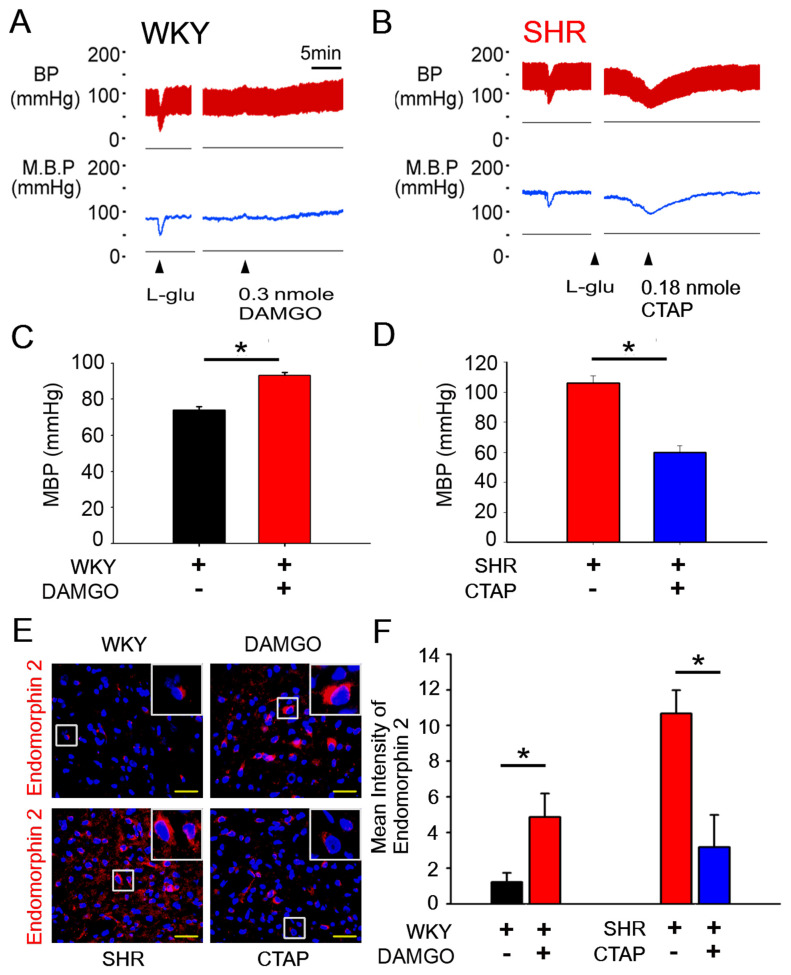
Activation of μOR in the NTS and cardiovascular regulation of WKY rats and SHRs. (**A**) Representative tracings demonstrating the cardiovascular effects of [D-Ala^2^, MePhe^4^, Gly^5^-ol]-enkephalin (DAMGO) (0.3 nmol) injected into the unilateral NTS of anesthetized WKY (▲ time of injection). (**B**) Representative BP recordings of an intra-NTS microinjection of the μOR antagonist CTAP in hypertensive SHRs (▲ time of injection). (**C**) Bar graphs showing the effects of 10-min treatment with DAMGO on the mean blood pressure (MBP) of anesthetized WKY. (**D**) Bar graphs showing the effects of 10-min treatment with DAMGO on the mean blood pressure (MBP) of anesthetized SHRs. (**E**,**F**) Representative red fluorescence images and the statistical analysis for Endomorphin-2-positive cells after DAMGO or CTAP treatment. The images were photographed at ×400 and 1000 magnification. The Mann–Whitney U-test was used for statistical analysis. Bar values are shown as mean ± SEM (*n* = 6); * *p* < 0.05 versus control.

Figure 3A shows that the unilateral microinjection of DAMGO, a μOR-specific agonist, into the NTS elicited a depressor effect as compared to the control (73.9 ± 2.03 mmHg and 93.3 ± 1.42 bpm, *p* < 0.05, paired *t* test; *n* = 3, Figure 3C). In addition, the endomorphin-2 level in the NTS of WKY was significantly increased (Figure 3E,F). Hypertensive SHRs had the highest endomorphin-1/2 level in their NTSs. Endogenous μ-opioids were blocked by a μOR-specific antagonist [D-Phe-Cys-Tyr-D-Trp-Arg-Thr-Pen-Thr-NH_2_] (CTAP). In Figure 3B, the BP of the hypertensive SHRs began to gradually decrease, reaching a minimum at approximately 30 min after intra-NTS CTAP microinjection (105.97 ± 5.05 mmHg and 59.77 ± 4.67 bpm, *p* < 0.05, paired *t* test; *n* = 4, Figure 3D) and showed significantly lower endomorphin-2 levels in the NTS of SHRs after CTAP injection (Figure 3E,F).

**Figure 4:**
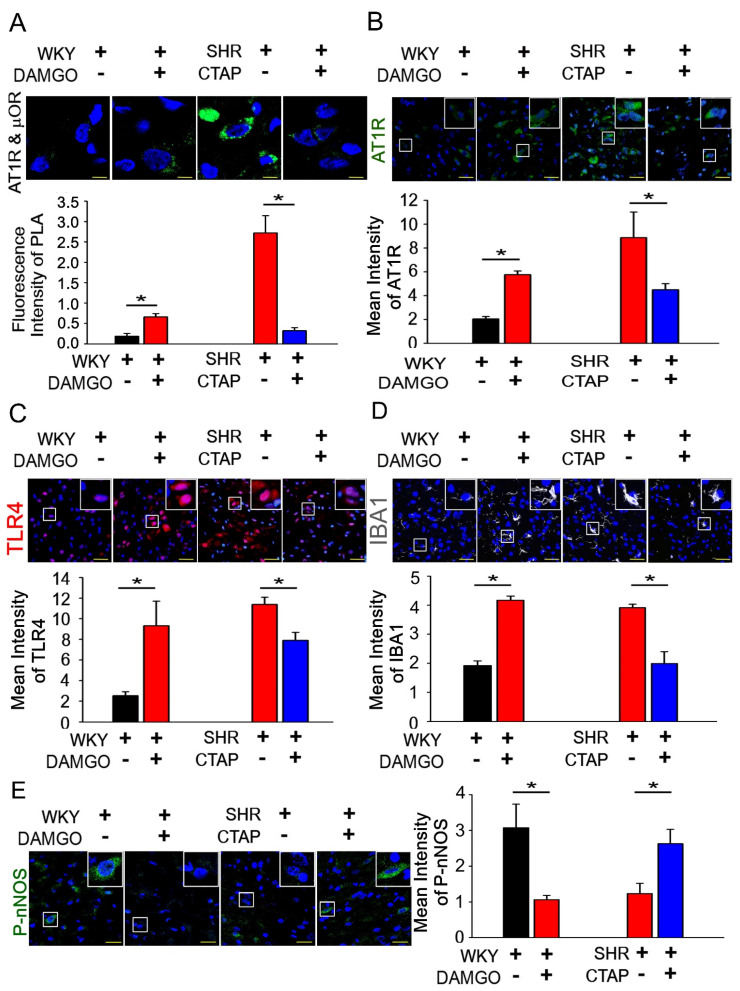
μOR elevates the formation of AT1R and μOR heterodimers, and induces the activation of microglia and the expression of TLR4 to impair the nNOS pathway in the NTS. (**A**) The in situ PLA (Proximity Ligation Assay) was used to confirm the formation of AT1R and μOR (μ-opioid receptors) heterodimers after intra-NTS DAMGO or CTAP microinjection. Green color indicates AT1R and μOR heterodimers; the nuclei were counterstained with DAPI. The images were photographed at 1000 magnification. (**B**–**E**) Representative fluorescence images of AT1R (green), TLR4 (red) microglial marker IBA-1 (white) and nNOS^S1416^ (green)-positive cells after intra-NTS DAMGO or CTAP microinjection. The images were photographed at ×400 and 1000 magnification. The Mann–Whitney U-test was used for statistical analysis. Bar values are shown as mean ± SEM (*n* = 6); * *p* < 0.05 versus control.

The regulation of BP by AT1R/μOR heterodimers was further investigated. The DAMGO-induced formation of AT1R/μOR heterodimers peaked at 10 min after DAMGO microinjection, and a reduction in CTAP was observed in the AT1R/μOR heterodimers (Figure 4A, *n* = 6). Furthermore, we found that AT1R, TLR4, activated microglial cells and nNOS^S1416^ phosphorylation were significantly increased in the DAMGO group compared to the control group. However, AT1R and TLR4 levels, activated microglial cells and nNOS^S1416^ phosphorylation were significantly lower in the CTAP group compared to the control group (Figure 4B,E, * *p* < 0.05, *n* = 6). These results indicate that the activation of μOR in the NTS may elevate AT1R to increase the activation of microglia and microglial TLR4.

## 4. Discussion

Despite recent advances in treatment options, approximately one-third of the adult population are affected by hypertension in the United States [33]. Essential hypertension, which is a rise in BP due to undetermined causes, includes 90% of all hypertensive cases and is estimated to cause 13% of all deaths [2]. Most patients with essential hypertension are obese and suffer from increased RAAS and AT1R activity, which exacerbates their risk for cardiovascular disease. A century of discoveries has established the importance of the RAAS in maintaining BP, fluid volume and electrolyte homeostasis through autocrine, paracrine and endocrine signaling. While research continues to yield novel functions for Ang II, angiotensin (1–7), angiotensin-converting enzyme inhibitors and Ang II receptor blockers, the gap between basic research and actual clinical application is yet to be solved [23].

Microglia is the major player in the brain innate immune system. Recent studies indicate that microglia and astrocytes in the brainstem and hypothalamus are involved in cardiovascular and metabolic events [34]. Our previous studies found that C-X3-C motif chemokine receptor 1 (CX3CR1) functions as a microglia biomarker, and that microglia suppresses the nNOS signaling pathway and promotes chronic inflammation in fructose-induced hypertension [15]. Recent studies also demonstrated that microglial activation in the paraventricular hypothalamic nucleus (PVN) and elevated proinflammatory cytokines (PICs) are found in Ang II-induced hypertension and SHR with high BP [35]. Previous reports showed that the blockade of brain microglia or the targeted depletion of activated microglia in the PVN attenuated Ang II-induced hypertension, decreased PVN cytokines and reduced cardiac hypertrophy, strongly demonstrating the important role of Ang II in microglial activation and the release of PICs in the pathogenesis of hypertension. Previous findings demonstrated that TLR4s modulated inflammatory responses implicated in the development of hypertension. As a prototypic TLR4 ligand, the acute administration of LPS activates microglia in the brain, and this response is attenuated by the blockade of AT1R [19]. In addition, Okechukwu et al. investigated the ability of Ang II to induce the release of the TLR4 ligand and high-mobility group protein 1 (HMBG1), and to augment TLR4 expression, which represents an alternative mechanism for Ang II stimulation of the innate system in the renal cells [20]. Therefore, we investigated the progression of hypertension after the activation of microglia and TLR4, the effects of TLR4 inhibitor TAK242 on SBP, the phosphorylation of nNOS^S1416^, and the activity of the microglia (Supplementary Figure S1). The present result showed that TAK-242-treated SHRs’ SBP was found to be significantly lower and the phosphorylation of nNOS^S1416^ was significantly higher in the NTS (Supplementary Figure S1A,B). Furthermore, TAK242-treated SHRs showed significantly reduced AT1R and TLR4 activation of microglial cells in the NTS (Supplementary Figure S1C,D). NO, the gas involved in sympathetic activity and blood pressure regulation in the NTS, was elevated through the inhibition of TLR4 microglia. Our results demonstrated that the increase in endogenous μ-opioid in the NTS induced the formation of μOR/AT1R heterodimers and the TLR4-dependent inflammatory pathway, which attenuated the NO-dependent depressor effect.

Oxidative stress and inflammation are essential for hypertension-induced renal injury. Toll-like receptors (TLR) are key regulators of the innate immune system, and TLR-4 deficiency reduces Ang-II-induced real injury and fibrosis via the attenuation of reactive oxygen species (ROS) production and inflammation in hypertensive kidneys [36]. Here, we show that AT1R-induced superoxide generation led to the activation of microglia and an increase in microglial TLR4, which were abolished by losartan treatment (Figure 2B–D). As previously noted, the regulation of Ang II and TLR4 involves the body–brain communication between afferent neural and humoral pathways that activate the central neural network to control cardiovascular function [36]. Neither RAS nor inflammatory mediators can contribute individually to the pathogenesis of hypertension; thus, the interactions between RAS components and inflammation mediators are likely synergistic [37]. Our findings concluded that the downregulation of AT1R-induced superoxide generation is associated with the activation of microglia and the expression of TLR4 in the NTS of SHR.

Opioids have been widely applied in clinics for centuries as one of the most potent pain relievers, but their abuse has deleterious physiological effects that are difficult to predict. In earlier studies, hypertension was characterized by pro- and antioxidant mechanisms [14], inflammatory disorders [15], GPCR heterodimers [16], and sympathetic/-parasympathetic tone imbalances [17]. Nevertheless, the mechanisms of the GPCRs involved in essential hypertension are not fully understood. A previous study showed that the formation of μOR/α_2A_-AR heterodimers in the NTS contributed to hypertension by disrupting the BP-lowering function of α_2A_-ARs [16]. Our previous results revealed that Ang II-induced stimulation generated the formation of AT1R and μOR heterodimers in the NTS, and downregulated the activity of the ERK1/2-RSK-nNOS pathway and the production of NO. In addition, opioids were previously shown to activate renin and ACE to increase the level of angiotensin II through either direct or indirect pathways [12]. A previous report showed that WKY that were ICV-infused with AngII showed increased BP, and that the AT1R and μOR heterodimers were also increased in the NTS [6].

**Figure 5:**
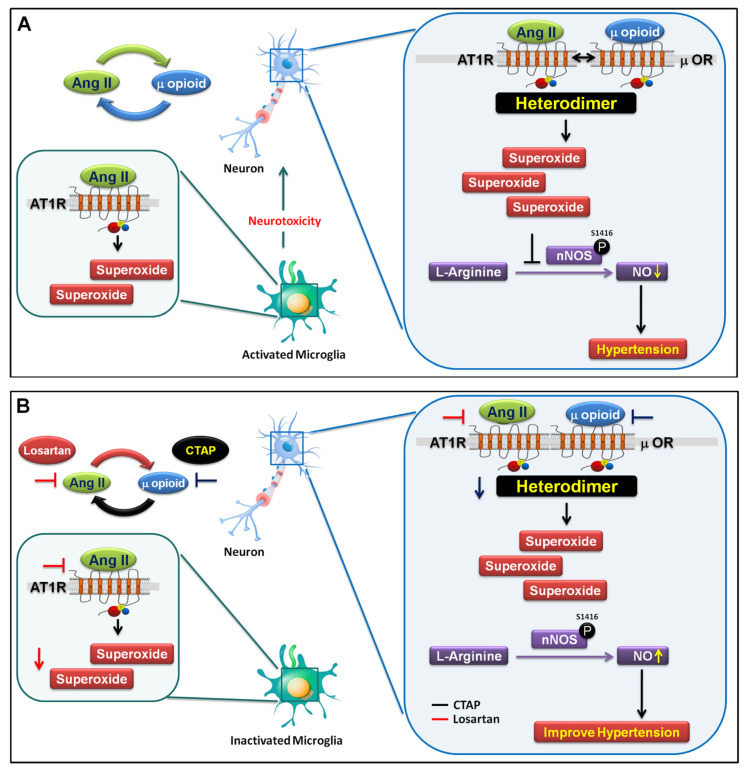
Proposed pathogenic mechanism for neurogenic hypertension. (**A**) The interaction of Ang II and μOR enhances the binding of Ang II to the AT1R receptor, activates the microglia and promotes the formation of AT1R-μOR heterodimers in the NTS, leading to superoxide production. This inactivates nNOS-derived NO, causing systemic elevations in blood pressure. (**B**) AT1R inhibitors (such as losartan) decrease superoxide production, abolish the activation of microglia and AT1R, and decrease TLR4 expression, which ultimately leads to improved hypertension (red line). Interestingly, μOR inhibitors (such as CTAP) decrease BP and abolish μOR-induced formation of AT1R and μOR heterodimers. CTAP also significantly inactivates the activation of microglia and AT1R, and reduces TLR4 expression, which can lead to an increase nNOS-derived NO levels, thereby improving hypertension (black line). In summary, this study shows how the interaction between Ang II and μOR enhances the binding of Ang II to AT1R, thereby causing microglia activation and inducing superoxide production, which, in turn, leads to neurotoxicity. Furthermore, the interaction of Ang II and μOR also enhances the formation of the AT1R and μOR heterodimers and inactivates nNOS-derived NO, leading to the development of progressive hypertension.

Microglia cells express the three subtypes of opioid receptors, μ, δ and κ [38]. Studies have shown that glial and neuronal μ opioid receptors have similar morphine binding affinities; however, glial cells express five times lower μ opioid receptors compared to neurons [39]. Wong et al. indicated that TLR9, not TLR2 or TLR4, plays a role in the morphine inhibition of *S. pneumoniae*-induced NF-κB activity in the early stage of infection [40]. He et al. also showed that TLR9 was required for the morphine-induced apoptosis of microglia through the p38 MAPK signaling pathway; in addition, he suggested that the inhibition of TLR9 and/or the blockage of µOR prevented opioid-induced brain damage [41]. Surprisingly, we observed that unilateral microinjection of a μOR-specific agonist, DAMGO, in the NTS led to the formation of the AT1R/μOR heterodimer, and that TLR4 expression was involved in the progression of hypertension. However, μOR specific antagonists [D-Phe-Cys-Tyr-D-Trp-Arg-Thr-Pen-Thr-NH_2_] (CTAP) have the reverse effect (Figure 4A,C). The μOR activated the AT1R, increased the number of microglial cells, and downregulated the phosphorylation of nNOS^S1416^ in the NTS (Figure 4B,D,E). These results indicate that μOR activation elevated AT1R to augment the activation of microglia and cause an increase in microglial TLR4, thereby leading to the progression of hypertension. These results also suggest that a reduction in GPCR stimulation through μOR is required to impair the formation of GPCR heterodimers and the depressor’s response. Given the role of Ang II in the maintenance of renal homeostasis, any novel inhibitor ought to possess improved selectivity for the targeting of pathogenic Ang II signaling to enable better hypertension treatment. Most importantly, we observed that the upregulation of endogenous μ-opioids in the NTS led to the interaction of Ang II and μOR, which promoted the binding of Ang II to AT1R, thereby activating the microglia and triggering superoxide production, and finally leading to neurotoxicity (Figure 5).

This study provides novel evidence that: (1) TLR4-dependent inflammatory levels were upregulated in the NTS of hypertensive SHRs; (2) AT1R inhibitors decreased BP and abolished TLR4-dependent inflammation in the NTS; (3) high μ-opioids levels triggered the formation of μOR/AT1R heterodimers in the NTS, which contributed to the development of hypertension; (4) the formation of the μOR/AT1R heterodimers enhanced TLR4-dependent inflammation, which impaired the NO-dependent depressor effect in the NTS; and (5) the TLR4-dependent inflammatory pathway also attenuated the NO-dependent depressor effect. Previous reports support these findings, indicating that μORs tend to form heterodimers with the formation of the α_2A_-ARs [42], and that μOR/α_2A_-AR heterodimers impair the function of α_2A_-ARs, which is consistent with our previous study [43]. G protein-coupled receptors (GPCRs) play an important role in drug therapy and are one of the largest families for drug targets. Similarly to other GPCRs, the μ-opioid receptor (µOR) carries out its function by stimulating the heterotrimeric G protein [44]. The formation of—or changes in—these ligands can alter dimer binding and receptor activation, and cause desensitization and trafficking, leading to pathophysiological processes. Further studies of these heterodimers, including AT1R and μOR or α2A-AR and μOR, will provide new insights into therapies against hypertensive conditions.

## 5. Conclusions

In conclusion, an abnormal increase in endogenous μ-opioid in the NTS not only induces a neurotoxicity cascade with enhanced Ang II binding to the AT1R receptor, and activates the microglia (which induces superoxide production), but also induces the formation of μOR/AT1R heterodimers and the TLR4-dependent inflammatory response, which attenuate the NO-dependent depressor effect. These findings deepen our understanding of μOR as a novel candidate for intervention in hypertensive conditions.

## Data Availability

All data generated or analysed during this study are included in this published article.

## Supplementary Materials

The following are available online at https://www.mdpi.com/article/10.3390/antiox10111784/s1, Figure S1: Downregulation of TLR4-induced neurotoxicity is associated with nNOSS1416 phosphorylation in the NTS of spontaneously hypertensive rats.

## Abbreviations

AT1R, angiotensin II type 1 receptor; Ang II, angiotensin II; AR, adrenergic receptors; α2A-AR, α2A-adrenergic receptor; BP, blood pressure; CNS, central nervous system; DAMGO, [D-Ala^2^, MePhe^4^, Gly^5^-ol]-enkephalin; GPCRs, G protein-coupled receptors; IBA1, ionized calcium binding adaptor molecule 1; PLA, Proximity Ligation Assay; NTS, nucleus tractus solitarii; NO, nitric oxide; μORs, μ-opioid receptors; RAAS, renin–angiotensin–aldosterone system; SHRs, spontaneous hypertensive rats; TLR4, toll-like receptor 4; WKY, Wistar-Kyoto rats.
