# Supplementary material for: μ-Opioid Receptor-Mediated AT1R–TLR4 Crosstalk Promotes Microglial Activation to Modulate Blood Pressure Control in the Central Nervous System
Source: Antioxidants (Basel). 2021 Nov 8;10(11):1784. doi: 10.3390/antiox10111784 (PMC8615018; doi:10.3390/antiox10111784)
Supplement: Supplementary file 1 [file antioxidants-10-01784-s001.zip › antioxidants-1347144-supplementary.pdf]

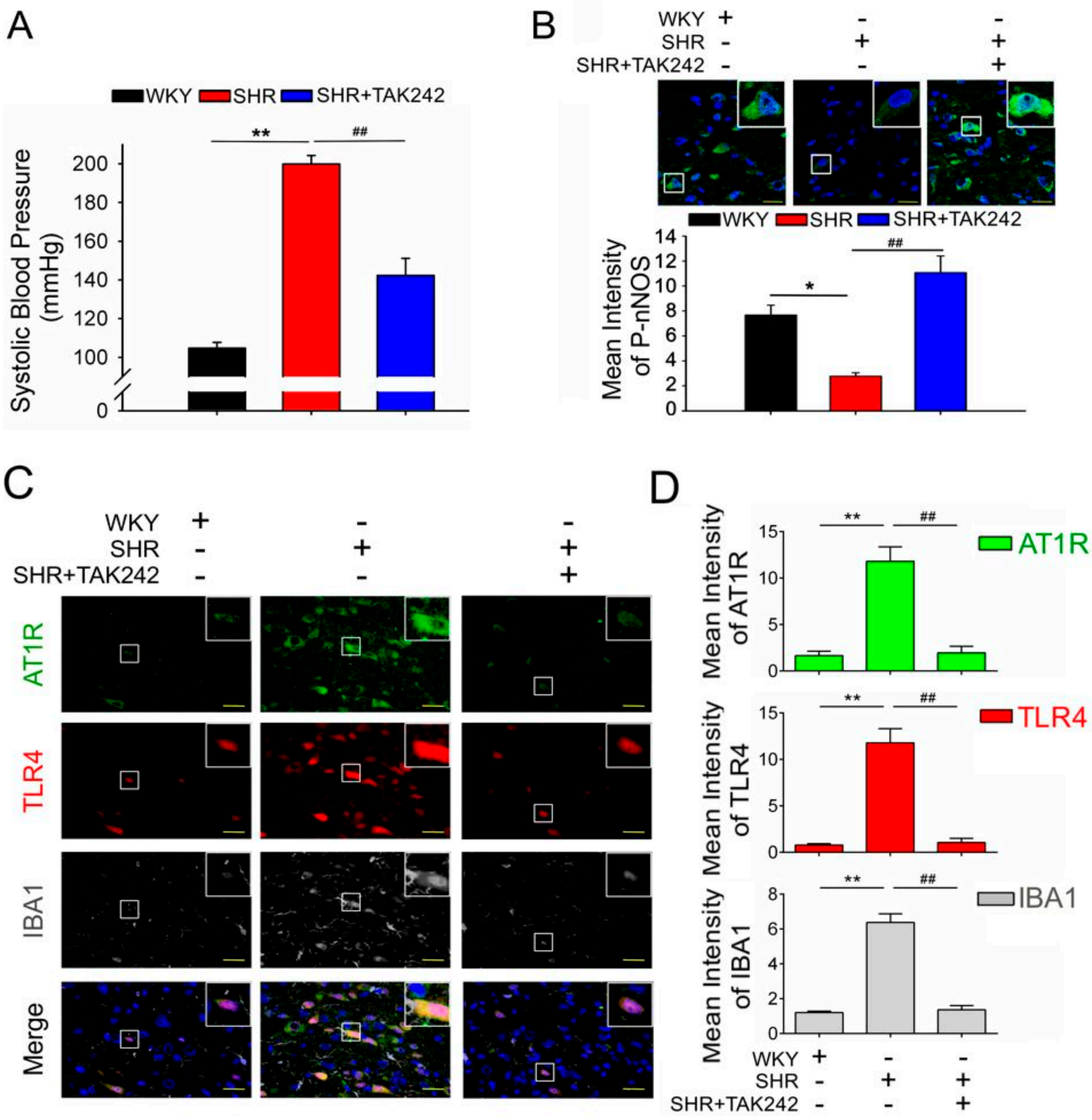

Supplementary Figure S1. Downregulation of TLR4-induced neurotoxicity is associated with nNOS^{S1416} phosphorylation in the NTS of spontaneously hypertensive rats. (A) SBP after TAK242 administration for 2 weeks. (B) Representative fluorescence images for nNOS^{S1416} (green)-positive cells in the NTS sections of WKY, SHRs, and SHRs treated with TAK242. One-way analysis of variance (ANOVA) with Scheffé post-hoc performed for statistical analysis. (C-D) Representative fluorescence images for AT1R (green), TLR4 (red) and microglial marker IBA-1 (white)-positive cells in the NTS sections of WKY, SHRs, and SHRs treated with TAK242. One-way analysis of variance (ANOVA) with Scheffé post-hoc was performed for statistical analysis. The values are presented as mean ± SEM. *P < 0.05, significantly different from 20-week-old WKY. #P <0.05 versus SHR. All data are presented as means ± SEM (n = 6 per group).
